# Sporadic hemangioblastomas are characterized by cryptic *VHL* inactivation

**DOI:** 10.1186/s40478-014-0167-x

**Published:** 2014-12-24

**Authors:** Ganesh M Shankar, Amaro Taylor-Weiner, Nina Lelic, Robert T Jones, James C Kim, Joshua M Francis, Malak Abedalthagafi, Lawrence F Borges, Jean-Valery Coumans, William T Curry, Brian V Nahed, John H Shin, Sun Ha Paek, Sung-Hye Park, Chip Stewart, Michael S Lawrence, Kristian Cibulskis, Aaron R Thorner, Paul Van Hummelen, Anat O Stemmer-Rachamimov, Tracy T Batchelor, Scott L Carter, Mai P Hoang, Sandro Santagata, David N Louis, Fred G Barker, Matthew Meyerson, Gad Getz, Priscilla K Brastianos, Daniel P Cahill

**Affiliations:** Departments of Neurosurgery, Massachusetts General Hospital, Boston, MA USA; Departments of Pathology, Massachusetts General Hospital, Boston, MA USA; Divisions of Neuro-Oncology, Massachusetts General Hospital, Boston, MA USA; Departments of Hematology/Oncology, Massachusetts General Hospital, Boston, MA USA; Cancer Program, Broad Institute, Cambridge, MA USA; Department of Pathology, Brigham and Women’s Hospital, Boston, USA; Departments of Neurosurgery, Seoul National University, Seoul, South Korea; Pathology, Seoul National University, Seoul, South Korea; Center for Cancer Genome Discovery, Dana Farber Cancer Institute, Boston, MA USA; Medical Oncology, Dana Farber Cancer Institute, Boston, MA USA

**Keywords:** Central nervous system, Hemangioblastoma, Deep sequencing, Somatic gene alterations, Von Hippel-Lindau gene, Hypoxia-inducible signaling

## Abstract

Hemangioblastomas consist of 10-20% neoplastic “stromal” cells within a vascular tumor cell mass of reactive pericytes, endothelium and lymphocytes. Familial cases of central nervous system hemangioblastoma uniformly result from mutations in the Von Hippel-Lindau (*VHL*) gene. In contrast, inactivation of *VHL* has been previously observed in only a minority of sporadic hemangioblastomas, suggesting an alternative genetic etiology. We performed deep-coverage DNA sequencing on 32 sporadic hemangioblastomas (whole exome discovery cohort n = 10, validation n = 22), followed by analysis of clonality, copy number alteration, and somatic mutation. We identified somatic mutation, loss of heterozygosity and/or deletion of *VHL* in 8 of 10 discovery cohort tumors. *VHL* inactivating events were ultimately detected in 78% (25/32) of cases. No other gene was significantly mutated. Overall, deep-coverage sequence analysis techniques uncovered *VHL* alterations within the neoplastic fraction of these tumors at higher frequencies than previously reported. Our findings support the central role of *VHL* inactivation in the molecular pathogenesis of both familial and sporadic hemangioblastomas.

**Electronic supplementary material:**

The online version of this article (doi:10.1186/s40478-014-0167-x) contains supplementary material, which is available to authorized users.

## Introduction

Hemangioblastomas comprise 1–2.5% of primary intracranial and 7-10% of spinal cord tumors, with a benign pathology notable for neoplastic “stromal” cells embedded in a dense network of vascular channels [1]. These tumors are largely diploid with few chromosomal abnormalities [2,3], and have served as the prototypic lesions in the study of genomic drivers of hypoxia-mediated metabolism in cancer [4]. Approximately 25% of hemangioblastomas are a result of Von Hippel-Lindau (VHL) disease, which is inherited in an autosomal dominant manner through a germline inactivating mutation of the tumor suppressor gene *VHL* on chromosome 3, with subsequent inactivation of the second allele by somatic mutation or gene loss in the neoplastic “stromal” cell. The clinical spectrum of VHL disease includes other neoplastic lesions, including retinal angiomatosis, pheochromocytoma and clear cell renal carcinoma.

Approximately 75% of hemangioblastomas are sporadic. *VHL* somatic mutations have been clearly identified in a fraction of sporadic tumors [2-10]. However, unlike in familial VHL disease, prior studies have indicated that the majority of sporadic hemangioblastomas do not have identifiable germline or somatic alterations in *VHL* (Table 1). This discrepancy stands in contrast to recent whole genome characterization of clear cell renal carcinomas, which have somatic mutations in the *VHL* gene in >80% of samples [11,12], focal deletion by loss of chromosome 3p26.1 in >90% [13], or epigenetic silencing by promoter methylation in 7% [13].

**Table 1:** **Findings from prior studies analyzing sporadic hemangioblastomas for somatic mutations, loss of heterozygosity or deletion of** ***VHL***

		**Somatic mutation**			**Loss of heterozygosity**			**Deletion**			**Biallelic** ***VHL*** **inactivation (%)**
**Reference**	**Patients**	**Method**	**Positive cases**	**Rate of somatic mutation (%)**	**Method**	**Positive cases**	**Rate of LOH (%)**	**Method**	**Positive cases**	**Rate of deletion (%)**	
Kanno et al. [6]	13	Sanger	3/13	23	NA	NA	NA	NA	NA	NA	NA
Oberstrass et al. [7]	18	Sanger	8/18	44	NA	NA	NA	NA	NA	NA	NA
Tse et al. [5]	5	Sanger	2/5	40	polymorphic markers	1/2	50	NA	NA	NA	10
Olschwang et al. [8]	18	Sanger	2/18	11	NA	NA	NA	NA	NA	NA	NA
Lee et al. [9]	20	Sanger	2/20	10	polymorphic markers	10/19	53	NA	NA	NA	11
Glasker et al. [3]	13	Sanger	3/13	23	polymorphic markers	5/13	38	NA	NA	NA	8
Gijtenbeek et al. [2]	16	Sanger	5/16	31	NA	NA	NA	CGH	11/16	69	19
Lemeta et al. [10]	11	NA	NA	NA	polymorphic markers	11/11	100	CGH	2/11	18	18

We considered two possibilities for this difference between the reported frequency of mutations in *VHL* between familial and sporadic hemangioblastomas. The first explanation is that sporadic cases of hemangioblastoma may develop via alterations in genes involved in hypoxia-sensing pathways other than *VHL* itself that also result in a histologically-identical phenotype of exuberant angiogenesis. A candidate gene for such an alteration is *TCEB1*, which encodes the required VHL binding partner elongin C and, in the recent genomic sequencing of clear cell renal carcinoma was found to be somatically mutated in the minority of cases that do not harbor *VHL* mutations [14]. A second explanation is that prior bulk sequencing techniques were technically limited in capturing the full extent of *VHL* inactivation. Neoplastic “stromal” cells comprise roughly 10-20% of the hemangioblastoma tumor cell mass with the remainder consisting of pericytes, vascular endothelium and other nucleated cells, such as lymphocytes, that are present in the vascular channels [15]. Given the relatively low purity of neoplastic cells, previous studies may not have detected the entire spectrum of alterations in *VHL*.

The emergence of next generation sequencing technologies has rapidly accelerated the discovery of somatic alterations in cancer genes, by exploiting greater depth-of-coverage to increase the sensitivity of detection within low purity samples [13,16-22]. Application of these techniques to hemangioblastomas can comprehensively examine the role of *VHL* across the spectrum of this disease. In addition, whether hemangioblastomas form from an embryologically arrested “hemangioblast” stem cell that subsequently differentiates into the discrete constituent cell types that support the architecture of this vascular tumor [1,23-25] is unclear. While developmental studies have shown a common precursor of the differentiated cell types (endothelial, stromal) found in the hemangioblastoma cell mass, whether neoplastic cells contribute to these different populations was not well-characterized. By analyzing the extent of genomic alterations and their subclonal and clonal status within the tumor mass of sporadic hemangioblastomas, this issue can be addressed. In this study, we characterize sporadic hemangioblastomas of cerebellum and spinal cord by whole exome sequencing and deep-coverage sequence analysis.

## Materials and methods

### Sample acquisition

This study was approved by the Institutional Review Board at the hospitals providing specimens. Written informed consent was obtained for all samples undergoing whole exome sequencing. The discovery and validation cohorts of sporadic hemangioblastomas involving the cerebellum and spinal cord were obtained from patients treated at Massachusetts General Hospital (MGH, Boston MA), Brigham and Women’s Hospital (BWH, Boston MA), and Seoul National University (South Korea). Patients were identified as likely sporadic hemangioblastomas based on (1) prior testing that was negative for germline *VHL* mutations by sequencing of the coding regions (exons 1–3), (2) lack of family history of hemangioblastomas or VHL disease, and/or (3) lack of other stigmata of VHL disease, such as renal cancer, pheochromocytoma, or retinal angiomas. Pathology was verified by the Departments of Pathology at MGH and BWH (DNL, SS and MA) and tumor rich areas were microdissected.

### Tumor sequencing

DNA was extracted from FFPE tumor specimens and matching peripheral blood samples using standard techniques (QIAamp, Qiagen) and quantified by PicoGreen dye (Invitrogen). Whole exome sequencing was performed by hybrid capture and next-generation sequencing as described previously [26]. In brief, DNA was fragmented by sonication (Covaris Inc., Woburn, MA) to 150 bp and further purified using Agencourt AMPure XP beads. 50 ng of size-selected DNA was then ligated to specific adaptors during library preparation (Illumina TruSeq, Illumina Inc., San Diego, CA). Each library was made with sample-specific barcodes and quantified by quantitative PCR (Kapa Biosystems, Inc., Woburn, MA), and two libraries were pooled to a total of 500 ng for exome enrichment using the Agilent SureSelect hybrid capture kit (Whole Exome_v1.1; Agilent Technologies, Santa Clara, CA). Several captures were pooled further and sequenced in one or more lanes to a final equivalent of two exomes per lane on a HiSeq 2500 system (Illumina Inc, San Diego, CA).

The validation cohort was sequenced for 560 cancer-associated genes and 39 translocations previously implicated in cancer (Additional file 1: Table S1). Briefly, DNA was sonicated to achieve an average fragment size of 250 bp, size selected and barcoded. Multiplexed pools were hybridized with biotinylated baits (Agilent SureSelect) designed to capture exonic sequences, including those of *VHL* and *ARID1B*. The captures were sequenced on the Illumina HiSeq 2500 in Rapid Run Mode.

Separately, Sanger sequencing of the TCEB1 Tyr79Cys hotspot, and the promoter and exon 1 of *VHL,* was performed on PCR amplified products from tumor genomic DNA. Chromatograms were manually reviewed to evaluate for the presence of somatic mutations, which may be represented as a minor allele.

### Mutation analysis

Somatic variant, germline variant, and small deletions and insertion calling was performed within the Firehose environment at the Broad Institute with the previously published MuTect, MapReduce, and Indelocator algorithms [27-30]. Pindel, an algorithm for detecting insertions and deletions (indels) [31], was used to detect indels in the coding regions of *VHL*. Because the samples were all obtained from formalin fixed, paraffin embedded (FFPE) pathology specimens, we applied a filtering algorithm to reduce the number of artifactual mutations introduced by FFPE [32]. The FFPE filter consists of two steps. First, the filter estimates the component of total sequencing error rate due to FFPE artifacts in a CpG island by scanning all reference C (or G) sites counting sites sequenced as T (or A) in the two possible read pair orientations. Second, the orientation of each C > T (or G > A) mutation is compared to a model of balanced read pair orientation (binomial with p = 0.5: no artifact) and a biased orientation characteristic of FFPE artifacts (binomial with p = 0.96), as FFPE-induced artifacts affect one of the DNA strands. The filter removes mutations consistent with the FFPE orientation bias to the degree where less than 1% of the surviving mutations in a given sample are consistent with FFPE artifacts. Significance of mutated genes was determined by MutSigCV [33].

### Copy number and rearrangement analysis

Segmented copy data was obtained using copy number ratios. These were calculated as the ratio of tumor read depth to the average read depth observed in a panel of normal samples. Subsequently we converted the copy ratio data to allelic ratio, which allows for detection of copy neutral LOH and gives another constraint on copy number alterations. ABSOLUTE [22] was used to estimate sample purity and ploidy and then calculate cancer cell fraction (inferred percentage of neoplastic nuclei that harbor a given mutation) and cell copy number of mutations and local DNA copy changes. Rearrangements were determined by the dRanger and BreakPointer algorithms [34].

### Loss of heterozygosity analysis of validation cohort

To assess whether *VHL* in our validation cohort was deleted or in copy neutral loss of heterozygosity, we examined the distribution of allele fractions of heterozygous mutations in the mix of reported somatic and germline variants. In a diploid state (ploidy = 2) germline heterozygous sites should be represented by 50% allele fraction. When samples gain or lose a copy, the allele fraction of those sites will shift in relationship to the purity of the tumor. In order to determine the copy state of each sample we identified peaks in the probability density function (pdf) of allele fractions:

$$ pdf(Chr3p)={\displaystyle \sum_m}pdf\left(af,\  alt(m)+1,\ ref(m)+1\right) $$

Where *af* is the true allele fraction between 0 and 1, and *alt* is the alternate allele counts, *ref* is the reference allele count, and *m* is each single nucleotide variant detected along chromosome 3p. Samples demonstrating allelic fractions with bimodal distributions were called positive for a deletion or copy neutral loss of heterozygosity.

For samples demonstrating allelic shift, tumor purity (P_T_) was further estimated from the allele fraction of the upper allele (χ_A_) in the two following scenarios.

1. if the LOH event represents a copy neutral event, then the tumor purity (P_T_) is given by the following equation:
  $$ {P}_T=2*\ {\chi}_A $$
2. if the LOH event was a result of haploidization, then the tumor purity is given by:

$$ {P}_T = \frac{2*\ {\chi}_A-1}{\chi_A} $$

### Immunohistochemistry

Five micron sections of the FFPE specimens from the discovery cohort were prepared and stained for HIF1-α (NB100-131, Novus Biologicals), VEGF (SC-152, Santa Cruz Biotechnology), or PDGFR-β (AB32570, Abcam) by protocols described in the product data sheets. IHC was reviewed and scored by MPH.

## Results

We performed whole exome sequencing (WES) of 10 sporadic hemangioblastomas for our discovery cohort and targeted sequencing of 22 sporadic hemangioblastomas for our validation cohort. Estimation of neoplastic stromal cell content was 10-30% at pathology review (Additional file 2: Figure S1). The average age of the patients in the combined cohorts was 53.3 years with 23 tumors resected from the cerebellum and 9 from the spinal cord (Table 2). WES of germline DNA from the discovery cohort did not reveal any *VHL* mutations, indicating that these patients do not have VHL disease and that the tumors we evaluated are bona fide sporadic hemangioblastomas. The exomes of tumor specimens in the discovery cohort were covered at a mean depth of 116× (Additional file 1: Table S2). We did not detect any rearrangements in the discovery cohort using previously reported algorithms, which can detect events that occur close to exons [34].

**Table 2:** **Baseline clinical characteristics of discovery and validation cohorts of sporadic hemangioblastomas sequenced in this study**

	**Discovery (n = 10)**	**Validation (n = 22)**
Average Age (IQR)	52 (44–61)	54 (44–63)
Location		
*Cerebellum*	8	15
*Spinal cord*	2	7
Gender		
*Male*	7	9
*Female*	3	13
Prior *VHL* testing	1	5
Additional VHL-related lesions	0	0

Interquartile range (IQR) for age of patients is represented in parentheses. Patients who had undergone prior clinical testing for germline *VHL* mutations are listed. No patients included in either cohort were noted to have canonical VHL-related lesions, including retinal hemangioblastomas, pancreatic lesions, pheochromocytomas, endolymphatic sac tumors, renal cysts or clear cell renal carcinoma.

**Figure 1:**
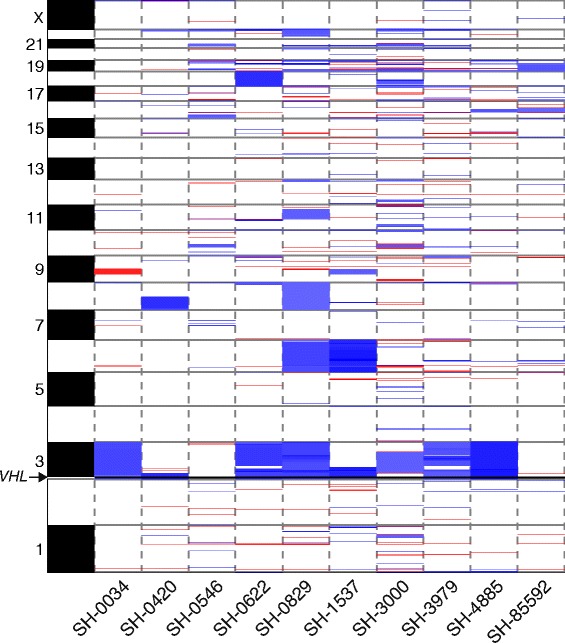
**Sporadic hemangioblastomas demonstrate recurrent losses of chromosome 3.** Segmentation of coverage and allelic data reveals recurrent deletion and LOH (blue) of chromosome 3. Each sample is represented as a column with evidence for amplifications denoted as red (allelic threshold 2.15-2.5) and deletions as blue (allelic threshold 1.5-1.85) along chromosomes which are represented as rows. The *VHL* locus is marked by the horizontal black line crossing chromosome 3p25 (indicated by arrow).

Copy number analysis of the WES data revealed recurrent loss of chromosome 3, but otherwise the genomes of tumors in the discovery cohort were diploid (Figure 1). By combining coverage ratio and allelic data to determine regions of somatic copy change and copy neutral loss of heterozygosity (copy neutral-LOH), we found focal hemizygous deletions of chromosome 3p resulting in loss of the *VHL* locus in 3 samples and hemizygous deletion of the entire chromosome 3p arm in 4 samples of the discovery cohort (Additional file 3: Figure S2, Additional file 1: Table S3). One sample was found to have focal copy neutral LOH of the *VHL* locus and an additional sample was found to have a copy neutral LOH of the entire chromosome 3p arm. Deletion of chromosome 8 was noted in 2 specimens and deletion of chromosome 6 in one specimen.

To identify somatic mutations in the WES data, a filter was first applied to exclude artifact mutations introduced by the processing of formalin-fixed, paraffin-embedded (FFPE) specimens, which are characterized by artificially induced C > T [32]. These artifact mutations are distinct from real CpG > TpG mutations, which dominate in most cancers. The FFPE filter accordingly removed 10 spurious mutations (Additional file 4: Figure S3).

**Figure 2:**
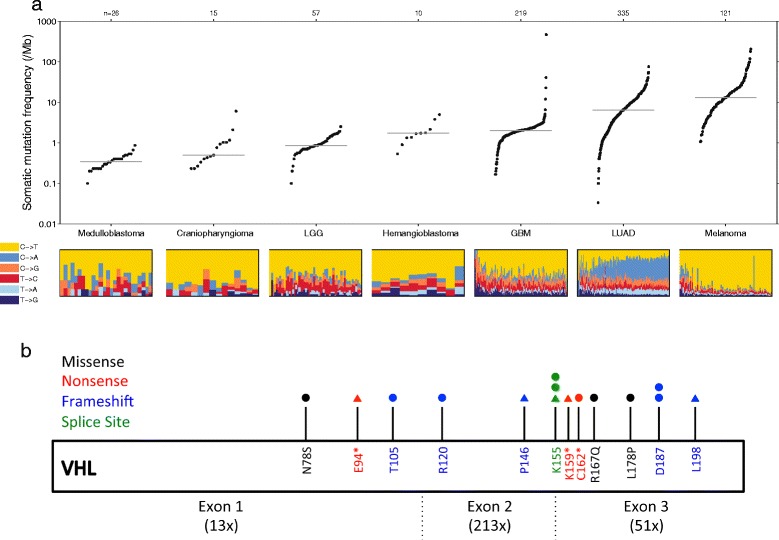
**Sporadic hemangioblastomas demonstrate low somatic mutation rates, but have recurrent mutations in** ***VHL*** **. (a)** Plot of the number of nonsynonymous mutations per megabase in hemangioblastomas in comparison to other primary tumors of the central nervous system, including medulloblastoma, craniopharyngioma, low grade glioma (LGG) and glioblastoma (GBM) [18]. For comparison, the mutation rate of lung adenocarcinoma and melanoma are displayed [24]. Each dot in this plot corresponds to a matched tumor-normal pair. The vertical position indicates the frequency of somatic mutations in that exome. The relative proportions of six different base-pair substitutions are indicated at the bottom. **(b)** Non-synonymous mutations in *VHL* observed in the discovery (triangle) and validation (circle) are illustrated. The average coverage depth of each exon for samples in the discovery cohort is indicated within parentheses.

The sequence data was then post-processed with MuTect [27] to identify true somatic mutations. The average overall mutation rate was 1.5/Mb with a total of 398 non-synonymous mutations and 118 synonymous mutations detected among the 10 samples (Additional file 1: Table S4). In comparison to other adult solid tumors, sporadic hemangioblastomas have a relatively low somatic mutation rate (Figure 2a), but one that is similar to other intracranial neoplasms with a tendency to local recurrence but not widespread dissemination, such as meningiomas (1.6/Mb) [26,35] and craniopharyngiomas (0.9/Mb) [19]. A total of 377 genes were found to have at least one non-synonymous mutation in the discovery set (Additional file 1: Table S4). Of these, *VHL* was mutated in 5 samples with an allele fraction ranging from 18-21% and cancer cell fraction from 76-100%, suggesting a clonal somatic mutation carried by the subpopulation of neoplastic “stromal” cells. *VHL* was the only gene found as statistically significant with respect to the false discovery rate by MutSigCV [27] (Figure 2b, q < 0.1), suggesting that no other gene is mutated at a similar rate.

Because exon 1 of *VHL* was covered at a lower mean depth (13×) than exons 2 (213×) and 3 (51×) (Figure 2b), the promoter region and exon 1 were separately PCR amplified and Sanger sequenced for the four specimens that did not demonstrate evidence for biallelic *VHL* inactivation. None of these specimens, for which exon 1 was initially covered at a mean of 7.4×, demonstrated somatic mutations in the promoter region or exon 1 of *VHL* by Sanger sequencing, although this method is not highly sensitive for detection of mutations with low allelic fractions. Of note, two missense mutations in *ARID1B* were identified in two separate hemangioblastomas, at amino acid positions 647 and 651, with predicted allelic fractions of 2-6% and cancer cell fraction of 8-32%, suggesting subclonality.

**Figure 3:**
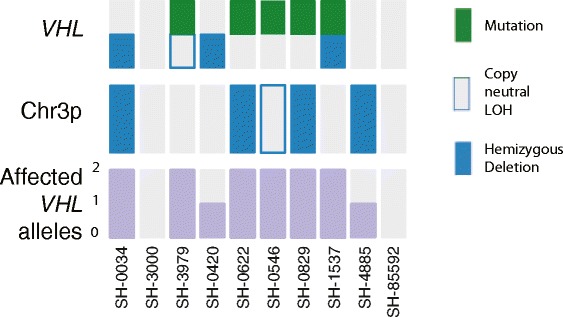
**Whole exome sequencing of sporadic cerebellar and spinal cord hemangioblastomas reveals recurrent VHL inactivation.** The *VHL* locus was noted to have non-synonymous mutations (green), copy neutral LOH (empty blue box), or hemizygous deletion (filled blue box) in 8/10 samples in the discovery cohort.

When mutations, deletions or LOH events involving the *VHL* locus are taken together, the discovery cohort of sporadic hemangioblastomas demonstrate biallelic inactivation of *VHL* in 6 of 10 cases and inactivation of at least a single allele of *VHL* in 8 of 10 cases (Figure 3).

**Figure 4:**
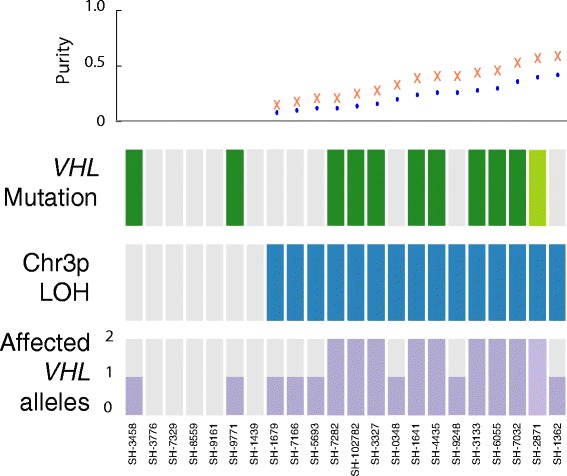
**High frequency recurrent somatic events involving the** ***VHL*** **locus are confirmed in a validation cohort of sporadic hemangioblastomas.** The validation cohort was noted to have non-synonymous mutations (green) or LOH (blue) of the *VHL* allele in 17/22 specimens. Sample SH-2871 was noted by Pindel to have 22-nucleotide tandem duplication in exon 3 (light green). A range for tumor purity in the validation cohort was imputed from LOH as a function of either a copy neutral event (blue dot) or haploidization (orange cross).

The validation cohort consisted of 22 sporadic hemangioblastomas, for which the exons of 560 genes (including *VHL* and *ARID1B*) and 39 translocations previously implicated in cancer were sequenced at an average depth of 182x. The validation cohort revealed LOH of chromosome 3p in 15 of the 22 samples (Additional file 5: Figure S4). Tumor purity was estimated for the validation specimen demonstrating LOH and ranged from 8-57% (Additional file 1: Table S5). *VHL* was sequenced at a mean depth of 87× in this cohort and eleven specimens were found to have non-synonymous mutations in *VHL* (Additional file 1: Table S6). One of these specimens was noted to have a frame shift 22-nucleotide tandem duplication in exon 3 within 8 codons of the stop codon. Of note, while the validation specimens were selected as representing sporadic hemangioblastomas by clinical information and germline testing for most, it is possible that up to 10% of these patients without matched blood may have germline *VHL* mutations [7,8]. Of specimens in the validation cohort that were found to have a non-synonymous mutation in *VHL*, 82% also had LOH (Figure 4). No non-synonymous mutations in *ARID1B* were detected in the validation set. In addition, no *TCEB1* Tyr79Cys mutations were detected in any of the 32 hemangioblastomas in this study.

**Figure 5:**
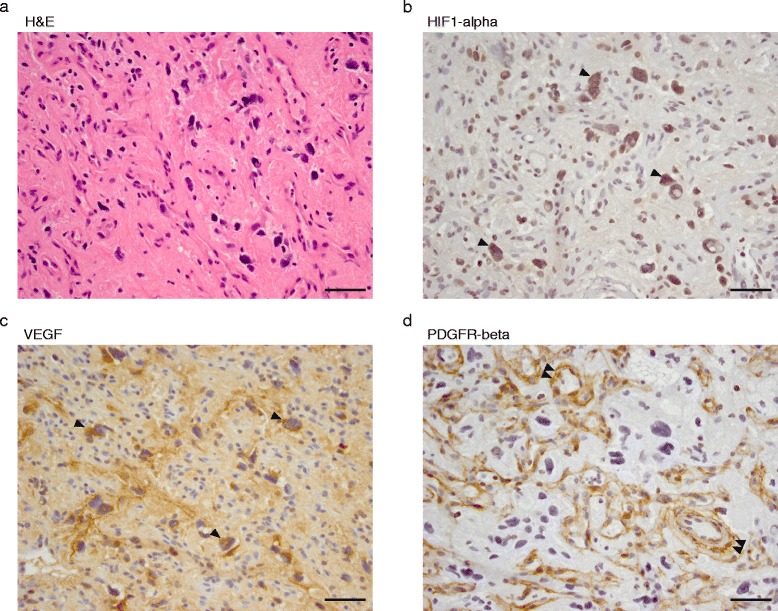
**Histologically distinct cell types in hemangioblastomas do not arise from a common ancestral clone.** “Representative images of sample SH-0622 acquired at 400x of **(a)** H + E and IHC for **(b)** HIF1-α, **(c)** VEGF, and **(d)** PDGFR-β reveal heterogenous cell types in this tumor characterized by a rich vascular network. Arrowheads indicate that the stromal cells demonstrate increased cytoplasmic staining for HIF1-alpha and VEGF, whereas the double arrowheads highlight PDGFR-beta protein restricted to vascular endothelium. Scale bar is 25 μm.

Prior studies have raised the possibility that hemangioblastomas arise from an embryologically arrested hemangioblast capable of differentiating into any of the cell types that comprise this vascular network, including pericytes, vascular endothelium, and even erythrocyte islands [1,23-25,36,37]. To assess the tumor cell fraction, we used ABSOLUTE, an algorithm which estimates tumor purity and ploidy allowing for calculation of cell copy number and cancer cell fraction of local DNA segments and mutations [22]. Similar algorithms were used recently for analyzing craniopharyngiomas, in which the fraction of neoplastic cells within the tumor bulk can be <10% [19]. Tumor purity of the discovery cohort determined by ABSOLUTE ranged from 9-49% (median 33%) (Additional file 1: Table S2), which corresponds to the neoplastic cell content of sporadic hemangioblastomas (Figure 5). This intercellular genomic heterogeneity suggests that the majority of nucleated cells in the tumor mass do not arise from a common ancestral clone, supporting the model that the tumor mass histology is a consequence of reactive angiogenesis resulting from paracrine signaling mediated by the VHL-deficient HIF1α-expressing “stromal” cells.

## Discussion

*VHL* mutations have been previously detected in sporadic hemangioblastomas [2-10]. Our study is the first to comprehensively set an upper bound on the frequency of other alterations in hemangioblastoma formation, and further confirm that *VHL* alterations can be detected in the vast majority of hemangioblastomas, establishing *VHL* inactivation as the central event in formation of this tumor. Based on the implications of prior work, these findings were one of the possible anticipated results in this prototypic tumor type, and were facilitated by next generation sequencing techniques. In addition, utilization of this technology allowed for quantification of allelic frequency, revealing the cell non-autonomous role of *VHL*-inactivated hemangioblasts/stromal cells driving and sustaining the tumor cell mass via recruitment of intermixed normal endothelial cells.

Biallelic *VHL* inactivation in hemangioblastoma results in insufficient degradation of HIF-1α, which then results in the inappropriate transcription of effectors normally expressed in hypoxic states, such as vascular endothelial growth factor (VEGF) or platelet derived growth factor B (PDGF B) [38]. Accordingly, the tumor cell mass is composed largely of non-neoplastic endothelial cells and surrounding pericytes, with only a minor component consisting of neoplastic “stromal” cells that actually bear driver mutations. The process of tumorigenesis is thus driven by a subpopulation of neoplastic cells that recruit and interact with diverse cellular types to form the tumor mass [39,40]. Genomic analyses that do not sufficiently account for such cell-type purity issues can incompletely characterize the spectrum of alterations within the neoplastic cellular compartment. Sporadic hemangioblastomas therefore offered an enticing opportunity to use the most current analytics in cancer genomics to uncover cryptic driver alterations, which may be present in only a minor subpopulation of the overall tumor mass, evading detection by prior bulk tissue analyses.

As predicted by the benign clinico-pathologic features, hemangioblastomas were notable for a relatively low frequency of mutations. Within this context of infrequent mutations and copy number alterations, we found a markedly high prevalence of recurrent somatic mutations (50%) and LOH of the gene locus on chromosome 3p (72%). Together, these alterations account for biallelic inactivation of *VHL* in 47% and an inactivating alteration in at least one allele in 78% of sporadic hemangioblastomas. We detected these alterations in specimens with tumor cell fractions as low as 9%. It is possible that we could have captured an even higher prevalence of *VHL* inactivation via somatic mutation or LOH with deeper sequencing across *VHL* in its entirety.

Prior studies have reported the presence of mutations in *VHL* in 10 to 44% of sporadic hemangioblastoma [4]. The frequency of aneuploidy of chromosome 3 has ranged from 18 to 69% in these sporadic tumors [2,3]. One study identified concurrent somatic mutation and LOH of *VHL* in only 1 of 13 sporadic hemangioblastomas [3] and another study identified missense mutations with deletion of *VHL* by comparative genomic hybridization in only 2 of 16 sporadic hemangioblastomas [2]. This is likely a consequence of lower *VHL* mutant allele fractions confined to the neoplastic “stromal” cells in sporadic hemangioblastomas, as compared to familial tumors that are characterized by heterozygous germline *VHL* alterations.

Mutations in *ARID1B*, which behaves as a tumor suppressor in other cancers [41-44], were detected in this study in 20% of the discovery cohort, a level that did not reach statistical significance by our analysis. Furthermore, the mutations were present with allelic fractions of only 2-6% and these lesions were found to be subclonal. Further analysis is needed to determine whether such subclonal variants are functionally important in supporting the development of sporadic hemangioblastomas [45,46].

*VHL* inactivation in the pathogenesis of sporadic hemangioblastoma mirrors the *VHL* alterations seen in benign renal cysts, which may serve as precursors of clear cell renal cancer [47]. These cysts are notable for biallelic *VHL* inactivation in renal tubular epithelial cells; whereas, clear cell renal cancer is additionally characterized by mutations in genes involved in the PTEN-mTOR pathway [13,14]. Similar to benign renal cysts, hemangioblastomas rarely demonstrate aggressive malignant features, and we did not detect additional clonal genetic drivers in hemangioblastomas, including other candidate genomic loci on chromosome 3p of *BAP1*, *SETD2*, and *PBRM1* implicated in clear cell renal carcinoma.

The analysis of tumor purity by ABSOLUTE also demonstrates that the extent of *VHL* inactivation in these tumors parallels “stromal” cell content estimated by IHC, rather than representing a homogenous genome that would characterize a tumor cell mass entirely derived from an ancestral hemangioblast clone. This important distinction lends further support to the model that stromal cells are genetically unique in *VHL* deficiency and stimulate reactive angiogenesis by recruiting normal endothelial cells via pseudo-hypoxia mediated paracrine signaling. This model contrasts with the alternate proposed pathway of an embryologically arrested “hemangioblast” ancestral clone that trans-differentiates into the various cell types contained in the tumor [24,25,36].

In summary, we find that inactivation of *VHL* is highly recurrent and characteristic of the majority of sporadic hemangioblastomas. In addition, we do not detect other common recurrent somatic mutations in these tumors. These data therefore confirm the central role of *VHL* inactivation in all hemangioblastomas, suggesting that treatment strategies for both familial and sporadic cases could be uniformly considered, as *VHL* loss in the neoplastic subpopulation likely orchestrates the architecture of these tumors. For example, VEGF inhibitors have been used in cases of multifocal familial hemangioblastomas with treatment responses [48,49]; their use in surgically-challenging sporadic disease should be considered. Advances in deep-coverage sequence analysis have thus resulted in a more complete understanding of the molecular basis of this canonical cancer type, and hold promise for the discovery of similarly cryptic drivers of tumorigenesis in other cancers with complex and diverse cellular composition.

## Conclusions

In this study, we performed whole exome sequencing of matched tumor-normal specimens of sporadic hemangioblastoma and found that *VHL* inactivation is more highly prevalent in this disease process than previously noted and that no other gene alteration was identified at a statistically significant level in these tumors. High depth sequencing was required to unmask the low allelic fraction of *VHL* mutations and copy number alterations found in the neoplastic “stromal” cells, which are present only in a fraction of cells comprising the overall tumor mass. The central role of *VHL* in the formation of sporadic hemangioblastomas suggests that the approach to these tumors can be universally considered as a single entity together with familial cases, which arise as a result of VHL disease.

## Additional files

Additional file 1: Table S1. List of exomes and translocations that were sequenced in the validation cohort. Coding regions of 560 genes and 39 translocations previously implicated in cancer were targeted in the analysis of this cohort. **Table S2.** Mean target coverage data in discovery cohort of tumor and matched normal specimen. Tumor purity represented as a percentage was estimated by ABSOLUTE. **Table S3.** Allelic copy ratio of sequenced exome segments in discovery cohort. **Table S4.** Composite table of somatic mutations detected in discovery cohort by whole exome sequencing. CCF column indicates fraction of neoplastic cells that harbor the mutation as determined by ABSOLUTE. **Table S5.** Loss of heterozygosity analysis of validation cohort. As described in Materials and methods, tumor purity was estimated from the fraction of the upper allele in the two following scenarios: (1) if the LOH event represents a copy neutral event or (2) if the LOH event was a result of haploidization. **Table S6.** Composite table of the somatic mutations detected in the validation cohort by targeted sequencing of 560 cancer associated genes (OncoPanel). Additional file 2: Figure S1. Representative histopathology of sporadic hemangioblastoma sequenced in this study. Hematoxylin and eosin stain of sporadic hemangioblastoma at 200× magnification (scale bar = 50 μm) reveals cellular heterogeneity consisting of pericytes, vascular endothelium, blood cells, and the neoplastic “stromal” cells which are cells with foamy cytoplasm representing 10-30% of the overall mass. Additional file 3: Figure S2. Segmentation of coverage and allelic data reveals recurrent deletion and LOH (blue) of the *VHL* locus in the discovery cohort. Darker shades of blue indicate increased copy loss. Each row represents a sample within the discovery cohort. The *VHL* locus along chromosome 3p is indicated by the red arrow. Additional file 4: Figure S3. Two dimension histogram of SNV read strand orientation bias (i_ffpe_F) vs. alternate allele counts (alt counts) in the C > T (or complement G > A) mutation mode. The color code on the right indicates the number of mutations falling into a given bin. The histogram at the bottom is a projection of all mutation to the i_ffpe_F axis and the histogram along the left is a projection onto alt allele counts. Real mutations are expected to have a symmetric distribution (binomial distribution with probablility 0.5 at each alt count) but there is a clear bias toward i_ffpe_F values approaching 1.0, particularly at the lowest allele counts. This bias is a result of the single strand damage characteristic of FFPE. The filter removes mutations occurring in the lower right section, with a threshold optimized to leave at most one percent of the surviving mutations arising from FFPE artifact [32]. Additional file 5: Figure S4. Sporadic hemangioblastomas in the validation cohort display recurrent loss of heterozygosity of chromosome 3p. The frequency of allele fractions of mutations on chromosome 3p were plotted and loss of heterozygosity was identified in 15 samples where allelic shift (vertical red and blue lines) was noted from the expected distribution centered at 0.5 (vertical black line).
