# Supplementary figures and images for: Sporadic hemangioblastomas are characterized by cryptic VHL inactivation
Source: Acta Neuropathol Commun. 2014 Dec 24;2:2. doi: 10.1186/s40478-014-0167-x (PMC4297409; doi:10.1186/s40478-014-0167-x)

Supplementary Figure 1

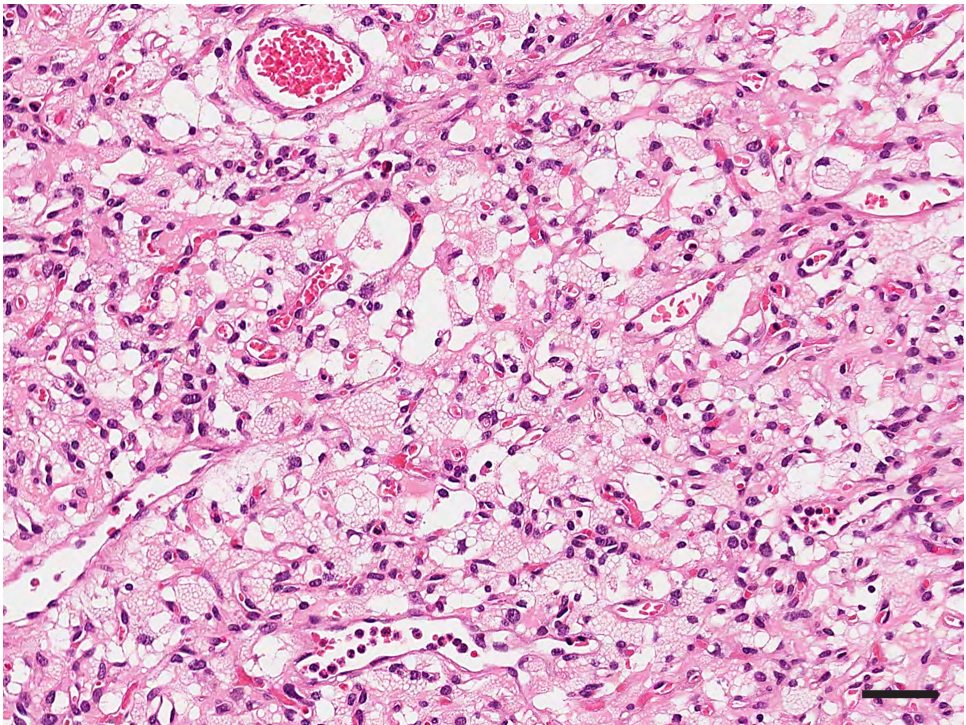

Supplement: Additional file 2: Figure S1. — Representative histopathology of sporadic hemangioblastoma sequenced in this study. Hematoxylin and eosin stain of sporadic hemangioblastoma at 200× magnification (scale bar = 50 μm) reveals cellular heterogeneity consisting of pericytes, vascular endothelium, blood cells, and the neoplastic “stromal” cells which are cells with foamy cytoplasm representing 10-30% of the overall mass. [file 40478_2014_167_MOESM2_ESM.pdf]

Supplementary Figure 2

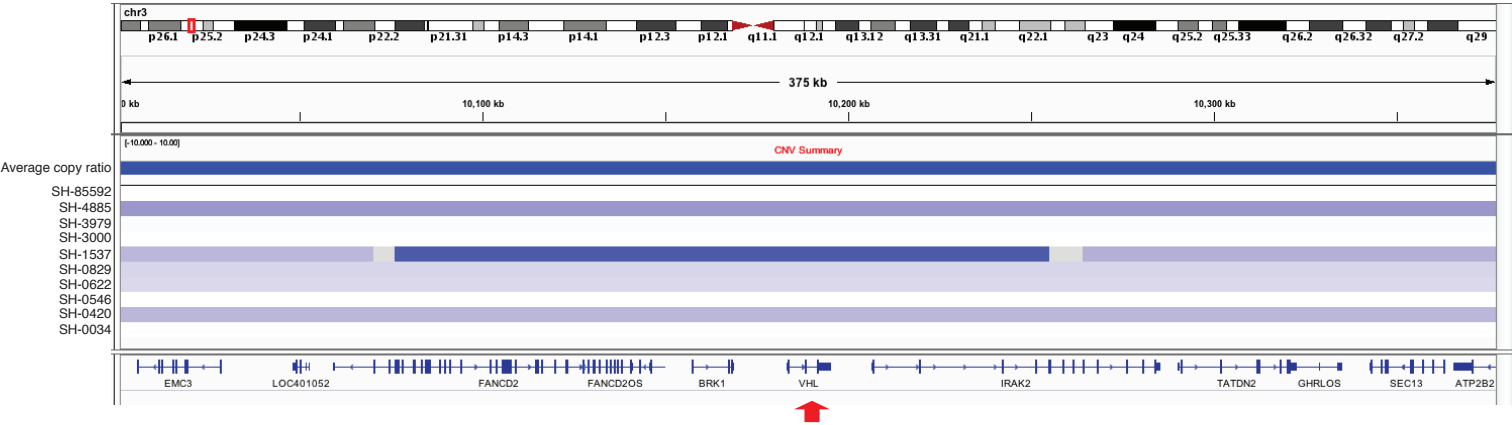

Supplement: Additional file 3: Figure S2. — Segmentation of coverage and allelic data reveals recurrent deletion and LOH (blue) of the VHL locus in the discovery cohort. Darker shades of blue indicate increased copy loss. Each row represents a sample within the discovery cohort. The VHL locus along chromosome 3p is indicated by the red arrow. [file 40478_2014_167_MOESM3_ESM.pdf]

Supplementary Figure 3

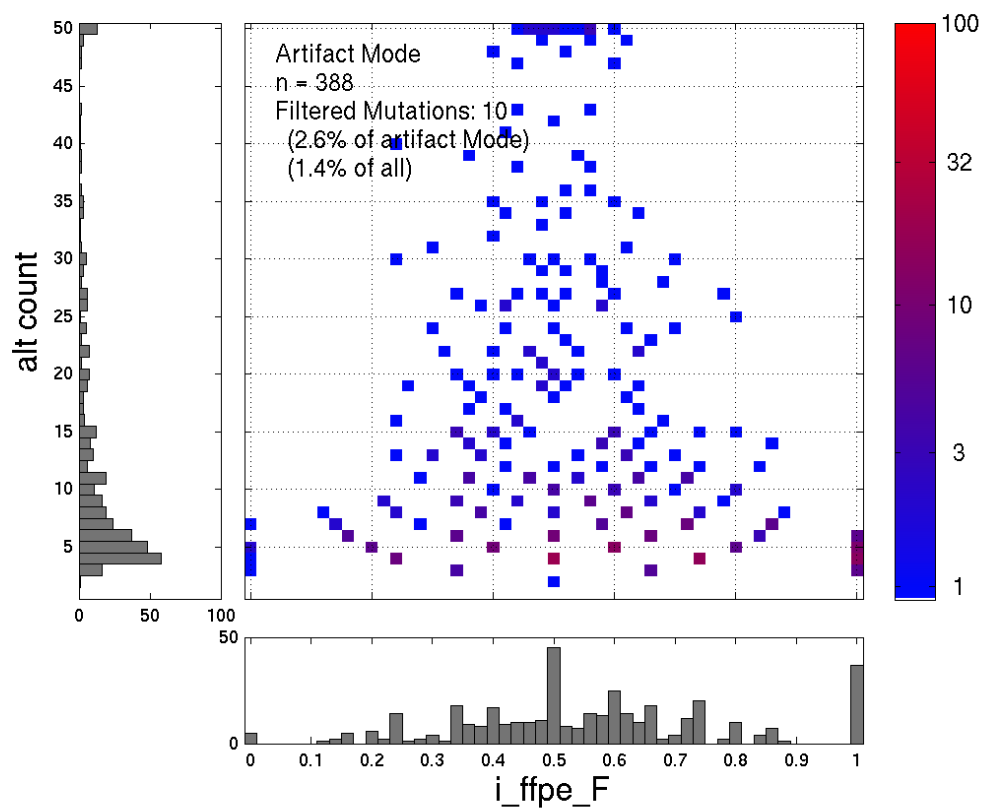

Supplement: Additional file 4: Figure S3. — Two dimension histogram of SNV read strand orientation bias (i_ffpe_F) vs. alternate allele counts (alt counts) in the C > T (or complement G > A) mutation mode. The color code on the right indicates the number of mutations falling into a given bin. The histogram at the bottom is a projection of all mutation to the i_ffpe_F axis and the histogram along the left is a projection onto alt allele counts. Real mutations are expected to have a symmetric distribution (binomial distribution with probablility 0.5 at each alt count) but there is a clear bias toward i_ffpe_F values approaching 1.0, particularly at the lowest allele counts. This bias is a result of the single strand damage characteristic of FFPE. The filter removes mutations occurring in the lower right section, with a threshold optimized to leave at most one percent of the surviving mutations arising from FFPE artifact [32]. [file 40478_2014_167_MOESM4_ESM.pdf]

Supplementary Figure 4

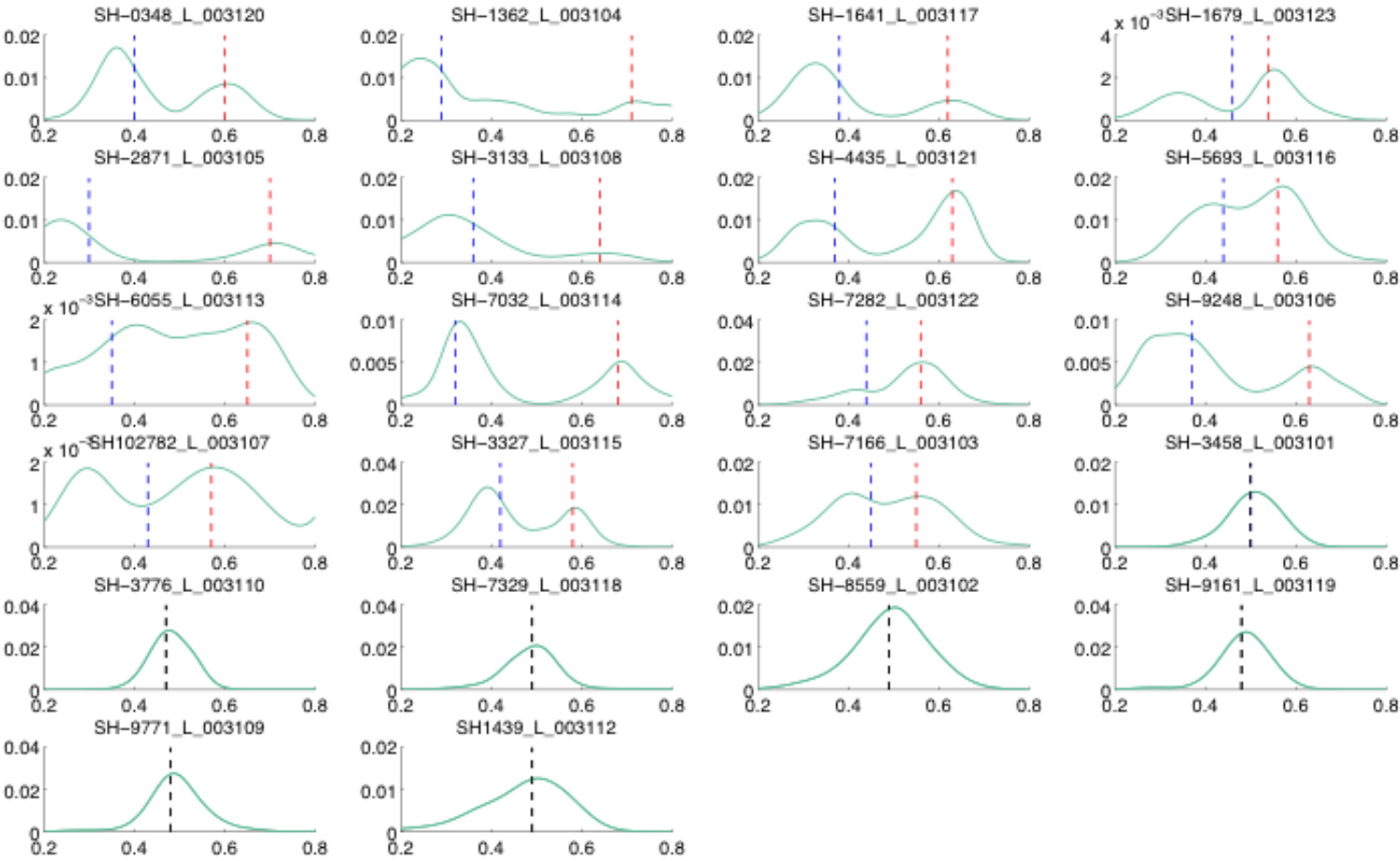

Supplement: Additional file 5: Figure S4. — Sporadic hemangioblastomas in the validation cohort display recurrent loss of heterozygosity of chromosome 3p. The frequency of allele fractions of mutations on chromosome 3p were plotted and loss of heterozygosity was identified in 15 samples where allelic shift (vertical red and blue lines) was noted from the expected distribution centered at 0.5 (vertical black line). [file 40478_2014_167_MOESM5_ESM.pdf]
